# The Dirichlet problem for the Jacobian equation in critical and supercritical Sobolev spaces

**DOI:** 10.1007/s00526-021-01931-9

**Published:** 2021-02-12

**Authors:** André Guerra, Lukas Koch, Sauli Lindberg

**Affiliations:** 1 University of Oxford, Andrew Wiles Building Woodstock Rd, Oxford, OX2 6GG UK; 2 Department of Mathematics and Systems Analysis, Aalto University, Aalto, P.O. Box 11100, 00076 Espoo, Finland

## Abstract

We study existence and regularity of solutions to the Dirichlet problem for the prescribed Jacobian equation, $\det D u =f$, where *f* is integrable and bounded away from zero. In particular, we take $f\in L^p$, where $p>1$, or in $L\log L$. We prove that for a Baire-generic *f* in either space there are no solutions with the expected regularity.

## Introduction

For $n>1$ let us take a bounded domain $\Omega \subset \mathbb {R}^n$ which is both smooth and uniformly convex. We consider the prescribed Jacobian equation, that is,

1.1 $$\begin{aligned} \det D u = f \quad in \Omega , \end{aligned}$$

which is a first-order fully non-linear underdetermined PDE. The equation (1.1) has a rich geometric flavour: formally, it can be written as $u^* (f \,d y)= \,d x$, where $u^*$ denotes the pull-back of the volume form $f(y) \,d y$ under *u*. Hence (1.1) can be read as asserting equivalence of volume forms over $\Omega$ with the same mass.

To be precise, we will consider a Dirichlet problem problem for (1.1):

1.2 $$\begin{aligned} {\left\{ \begin{array}{ll} \det {D u}=f &{} in \Omega ,\\ u=id &{} on \partial \Omega . \end{array}\right. } \end{aligned}$$

Here $f:\Omega \rightarrow \mathbb {R}$ is integrable and satisfies the compatibility and non-degeneracy conditions

1.3 $$\begin{aligned} \frac{1}{|\Omega |}\int _\Omega f(x)\,d x = 1 \qquad and \qquad \inf _\Omega f\ge c> 0. \end{aligned}$$

Whenever *f* is smooth or, more generally, Hölder continuous, it is natural to look for solutions which are at least $C^1$ and thus (1.2) can be understood classically. Under these assumptions, there is a very complete well-posedness theory going back to the seminal works of Moser [41] and Dacorogna–Moser [17], see also the book [15] and the references therein. An alternative approach to this problem was taken in [13], where the well-posedness theory for (1.2) is derived from Caffarelli’s regularity theory [11, 12] for the Monge–Ampère equation.

A recurring theme in the modern theory of PDE is to study well-posedness in spaces of weak solutions. In this paper we are interested in studying Sobolev solutions of (1.2) when $f\in L^p(\Omega )$ and we will focus, in particular, on the case where the solutions are in a critical or supercritical Sobolev space. In contrast with the rich classical theory, almost nothing is known in this setting. Indeed, and since the Jacobian is an *n*-homogeneous polynomial in $D u$, we have the following natural question:

### Question 1

Let $1\le p<\infty$ and assume *f* satisfies (1.3).

(i) If $p>1$, $f\in L^p(\Omega )$, does (1.2) have a solution $u\in W^{1,np}(\Omega ,\mathbb {R}^n)$? See [52, Question 3].
(ii) If $p=1$, $f\in L\log L(\Omega )$, does (1.2) have a solution $u\in W^{1,n}(\Omega ,\mathbb {R}^n)$? See [28, page 206].

The case $p=\infty$ is ruled out from Question 1 and it was addressed in [10, 39], see also [50], where it was shown that in this case the answer is negative, even when *f* is continuous. Partial positive results were obtained in [46]. The case $p=1$, where one must replace $L^1(\Omega )$ with $L\log L(\Omega )$ [28], also deserves special attention. Indeed, the Jacobian benefits from an improved integrability, first noticed by Müller [43] and then generalised in [14]; an estimate up to the boundary was obtained in [28] and, in the planar case, a beautiful sharp version can be found in [3]. As noticed in [25], this improved integrability is essentially a by-product of the weak continuity of the Jacobian, see Sect. 2.2 for more details. We note that the special behaviour of the Jacobian at the endpoints $p=1$, $p=\infty$ is not unexpected: for the divergence equation, which is a linearisation of the prescribed Jacobian equation, one also has non-existence of solutions when $f\in L^1$ or $f\in L^\infty$, due to cases [40, 45], see also [19, 34] and [7, §2].

The answer to both parts of Question 1 is negative in a rather strong sense. Indeed, let us consider for $1\le p<\infty$ the complete metric spaces

$$\begin{aligned} \begin{aligned} X_p&\equiv \left\{ f\in L^p(\Omega ): f satisfies (1.3) \right\} , \qquad for p>1\\ X_1&\equiv \left\{ f\in L\log L(\Omega ): f satisfies (1.3) \right\} . \end{aligned} \end{aligned}$$

We then have:

### Main Theorem

Fix $0<c<1$ and $1\le p<\infty$. Let $\Omega \subset \mathbb {R}^n$ be a smooth uniformly convex domain. There is $f\in X_p$ such that the Dirichlet problem (1.2) has no solution ${u\in W^{1,np}(\Omega ,\mathbb {R}^n)}$. In fact, for a Baire-generic $f\in X_p$, (1.2) has no solution in the space $\bigcup _{n\le q,\, p<q} W^{1,q}(\Omega ,\mathbb {R}^n).$

We briefly describe the proof of the main theorem. Assume for simplicity that $\Omega =B$ is a ball. The starting point of our proof is the observation that if $f\in L^p(B)$ is a radially symmetric function then the unique spherically symmetric solution of (1.2) is, in general, in $W^{1,p}\backslash W^{1,q}$ for any $q>p$, see Proposition 3.1. Hence we first show that any $L^p$ function admits perturbations which are radially symmetric in a small neighbourhood of $\partial B$ and such that the $W^{1,q}$-norm of the symmetric solutions in that neighbourhood is unbounded. The crucial step of the proof is to show that, for these perturbations, the symmetric solutions have comparable *q*-Dirichlet energy to that of the energy-minimiser: hence we deduce that the $W^{1,q}$-norm of *q*-energy-minimal solutions [see equation (4.2)] is unbounded at every $f\in X_p$. The weak continuity of the Jacobian, together with an application of the Baire Category Theorem, then concludes the proof.

It will be clear from the proof that the boundary condition $$u=id $$ on $$\partial \Omega $$ in (1.2) can be relaxed to the requirement that $$u(x) \in \partial \Omega $$ for $$\mathscr {H}^{n-1}$$-a.e. $$x\in \partial \Omega $$. Under this generalised boundary condition, our arguments also deal easily with the case where the compatibility condition in (1.3) is replaced by the condition $$\frac{1}{|\Omega |}\int _\Omega f(x)\,d x=k$$ for some fixed $$k\in \mathbb {N}$$. In general, the number *k* gives an essential upper bound on the multiplicity function of solutions.

Our main theorem raises the following question, which we do not address here:

### Question 2

For $n\le p=q<\infty$ and $f\in X_p$, does (1.2) have a solution $u\in W^{1,q}(\Omega ,\mathbb {R}^n)$?

Question 2 is natural from the viewpoint of the regularity theory for optimal transport maps. In Optimal Transportation, the underdetermined equation (1.1) is usually turned into a determined elliptic equation by imposing the constraint $u=D \phi$ for some convex scalar function $\phi$: thus $\phi$ satisfies the Monge–Ampère equation $\det D ^2 \phi =f$, see [8, 18, 49]. The relation between the prescribed Jacobian equation and the Monge–Ampère equation is analogous to that of their linear counterparts, the divergence equation and the Poisson equation, see Table 1. Moreover, the estimates for the Monge–Ampère equation scale as in these linear problems, which is precisely the scaling in Question 2.

However, the failure of a certain estimate for the determined problem does not imply that the same happens for the underdetermined problem, as shown by Bourgain–Brezis [7]. Surprisingly, even in the linear setting, *solutions of the determined problem do not have optimal regularity for the underdetermined problem*! This is also the case for the non-linear problem (1.1), at least if we only require $\inf _\Omega f\ge 0$. Indeed, at [52, page 293], Ye gives an example in $\Omega = B \subset \mathbb {R}^2$ such that (1.2) has a smooth solution but the corresponding solution of the Monge–Ampère equation is only $C^{1,1}$ regular.

**Table 1:** Particular solutions to the underdetermined problem are obtained by taking $u=D \phi$ for $\phi$ a solution of the corresponding determined problem

	Linear	Non-linear
Determined	$\triangle \phi = f$	$\det D ^2 \phi = f$
Underdetermined	$div \, u = f$	$\det D u = f$

In the case $n^2/(n+1) \le q < n$, Müller has posed an outstanding problem about the range of the *distributional Jacobian* $u \mapsto Det D u$, see [44, page 659]. In [35] it was shown that for any $1<q<n$ and any $f\in L^{q/n}(\Omega )$ there is a *pointwise solution* $u\in W^{1,q}(\Omega ,\mathbb {R}^n)$ of $J u = f$ and one may prescribe any boundary datum $g \in W^{1-1/q,q}(\partial \Omega )$. The free choice of the boundary datum precises the well-known fact that the pointwise Jacobian loses its geometric content when $q < n$. Nevertheless, for $q \ge n$, as in this paper, the distributional Jacobian and the pointwise Jacobian agree. We do not discuss distributional Jacobians further, instead referring the reader to [1, 4, 9, 42].

To conclude the introduction we briefly discuss the motivation for the work initiated on this paper, which is twofold. Firstly, the Jacobian plays an important role in Continuum Mechanics, specially in nonlinear elasticity, see e.g. [5, 16, 20, 28]. This is not surprising since, for a Sobolev map, positivity of the Jacobian implies a weak form of local invertibility [21]. Secondly, in $\mathbb {R}^n$, there is an intimate connection between the Jacobian equation, commutator estimates and factorization theorems in Hardy and $L^p$ spaces [29, 30, 36]; an outstanding open problem in the area is to determine whether the Jacobian is onto the Hardy or $L^p$ space [14, 31, 38]. Positive partial results can be found in [14, 30, 36, 37] and negative ones in [38]. We refer the reader to our forthcoming work [23, 24] for further details and progress towards the possible non-surjectivity of the Jacobian.

## Notation and background results

In this section we gather a few preliminary results for the convenience of the reader.

The set $\Omega$ will always denote a bounded smooth domain in $\mathbb {R}^n$. Moreover, in Sect. 4, we will further assume that $\Omega$ is *uniformly convex*, in the sense that the second fundamental form of $\partial \Omega$ is uniformly positive.

We will often write $J u\equiv \det D u$. As is customary, the symbols $a \approx b$ and $a\lesssim b$ are taken to mean that there is some constant $C>0$ independent of *a* and *b* such that $C^{-1} a \le b \le C a$ and $a\le C b$, respectively. We denote by $\mathbb S^{n-1}$ the unit sphere in $\mathbb {R}^n$ and, for $r<R$, we write

$$\begin{aligned} \mathbb {A}(r,R)\equiv \{x\in \mathbb {R}^n: r<|x|<R\}, \qquad \mathbb {A}[r,R]\equiv \{x\in \mathbb {R}^n: r\le |x|\le R\}. \end{aligned}$$

Here $|\cdot |$ denotes the Euclidean norm of a vector in $\mathbb {R}^n$; for a matrix $A\in \mathbb {R}^{n\times n}$, we also write |*A*| for its operator norm. We represent by $\mathscr {L}^n$ the *n*-dimensional Lebesgue measure, by $\mathscr {H}^{n-1}$ the $(n-1)$-dimensional Hausdorff measure and we write $\omega _n \equiv \mathscr {L}^n(B_1(0))$.

### Mappings of finite distortion

In this section we gather a few results from the theory of mappings of finite distortion, which is detailed in [26, 32], see also [2] for the planar case.

#### Definition 2.1

Let $u\in W^{1,1}_loc (\Omega ,\mathbb {R}^n)$ be such that $0 \le J u\in L^1_loc (\Omega )$. We say that *u* is a *map of finite distortion* if there is a function $K:\Omega \rightarrow [1,\infty ]$ such that $K<\infty$ a.e. in $\Omega$ and

$$\begin{aligned} |D u(x)|^n \le K(x)\, J u(x) \quad for a.e.\ x in \Omega . \end{aligned}$$

If *u* has finite distortion, we can set $K u(x) = \frac{|D u|^n}{J u(x)}$ if $J u(x)\ne 0$ and $K u(x)=1$ otherwise; this function is the (optimal) *distortion* of *u*.

We state some basic properties of the topological degree, referring the reader to [21] for further details.

#### Lemma 1

For $u\in C^0(\overline{\Omega }, \mathbb {R}^n)$ and $x\in \mathbb {R}^n\backslash u(\partial \Omega )$, the *topological degree* of *u* at *x* with respect to $\Omega$, denoted by $deg (x,u,\Omega )$, has the following properties:

(i) $$deg (\cdot ,u,\Omega ):\mathbb {R}^n\backslash u(\partial \Omega )\rightarrow \mathbb {Z}$$;
(ii) if $u=v$ on $\partial \Omega$, then $deg (\cdot , u,\Omega )=deg (\cdot , v,\Omega )$ on $\mathbb {R}^n\backslash u(\partial \Omega )$;
(iii) if $x\not \in u(\partial \Omega )$ and $deg (x,u,\Omega )\ne 0$ then $x\in u(\Omega )$.

For our purposes, the following classical result [51] is particularly relevant:

#### Theorem 2.2

Let $u\in W^{1,n}(\Omega ,\mathbb {R}^n)$ be a map of finite distortion. Then *u* has a continuous representative with the Lusin (N) property.

Recall that a map has the *Lusin (N) property* if it maps $\mathscr {L}^n$-null sets to $\mathscr {L}^n$-null sets. Theorem 2.2 gives no improvement in the supercritical case, since the continuous representative of a map $u\in W^{1,p}$, $p>n$, has the Lusin (N) property.

Theorem 2.2 shows that solutions of (1.2) satisfy the change of variables formula:

#### Corollary 2.3

Let $u\in W^{1,n}(\Omega ,\mathbb {R}^n)$ be a solution of (1.2) and (1.3). Then

$$\begin{aligned} \mathscr {L}^n(u(E)) = \int _E f \,d x\qquad for all measurable sets E\subseteq \Omega . \end{aligned}$$

#### Proof

The non-degeneracy assumption (1.3) implies that *u* is a map of finite, and even integrable, distortion. Hence, by Theorem 2.2, the coarea formula applies, see for instance [21, Theorem 5.23]: if $\mathcal {N}$ is the multiplicity function $\mathcal {N}(y,u,E)\equiv \#\{x\in E: u(x)=y\}$, then

2.1 $$\begin{aligned} \int _{u(E)} \mathcal N(y,u,E) \,d y= \int _E f\,d x, \qquad for all measurable sets E\subseteq \Omega . \end{aligned}$$

Moreover, *u* is injective a.e., that is $\mathcal N(y,u,\Omega )\le 1$ for a.e. $y\in \mathbb {R}^n$: it suffices to apply (2.1) with $E=\Omega$, recalling that $\int _\Omega f\,d x=|\Omega |$ by (1.3). Hence the conclusion follows from (2.1).

Although this will not be needed for the proof of the Main Theorem, it is worthwhile mentioning that solutions of (1.2) in a sufficiently good Sobolev space are homeomorphisms. The sharp statement follows for instance from the main result in [27]:

#### Theorem 2.4

Let $u\in W^{1,n}(\Omega ,\mathbb {R}^n)$ be a continuous map with distortion in $L^{n-1}(\Omega )$. If there is a compact set $K\subset \Omega$ such that $u|_{\Omega \backslash K}$ is discrete then *u* is open and discrete.

Recall that a map is *discrete* if the preimage of every point is locally finite. A counterexample due to Ball [5] shows that the conclusion of Theorem 2.4 does not hold if the distortion is in $L^q(\Omega )$ for $q<n-1$. We then deduce:

#### Corollary 2.5

Let $u\in W^{1,n(n-1)}(\Omega , \mathbb {R}^n)$ be a solution of (1.2), (1.3). Then $u\in Hom (\overline{\Omega }, \overline{\Omega })$.

#### Proof

It follows from our assumptions that the distortion of *u* is in $L^{n-1}(\Omega )$. Consider a sufficiently small neighbourhood $\Omega _\delta$ of $\Omega$ and extend *u* to be the identity in $\Omega _\delta \backslash \Omega$. Theorem 2.4, applied in $\Omega _\delta$, shows that $u|_\Omega$ is open and discrete and it follows that $u\in Hom (\overline{\Omega },u(\overline{\Omega }))$, see e.g. [26, Theorem 3.27]. Moreover, $u(\overline{\Omega })=\overline{\Omega }$. Indeed, it suffices to prove that $u(\Omega )\subseteq \Omega$, since

2.2 $$\begin{aligned} u(\overline{\Omega })=u(\Omega ) \sqcup u(\partial \Omega )= u(\Omega )\sqcup \partial \Omega \end{aligned}$$

where the unions are disjoint since *u* is a homeomorphism, and $\Omega \subseteq u(\Omega )$ by Lemma 1. Suppose that there is $x\in \Omega$ such that $u(x)\in \mathbb {R}^n \backslash \overline{\Omega }$. Take $y\in \Omega$ such that $u(y)\in \Omega$ and consider a continuous path in $\Omega$ joining *x* and *y*. The image of such a path under *u* must cross $\partial \Omega$ somewhere, which contradicts (2.2). $\square$

In fact, a simple argument using the change of variables formula shows that the inverse map $u^{-1}$ is in $W^{1,n}(\Omega ,\Omega )$. Note that the situation in the planar case is particularly pleasant, since then $n-1=1$. Other results in the direction of Corollary 2.5 can be found in [5, 48].

### Improved integrability of the Jacobian

In this section we recall standard facts concerning the improved integrability of the Jacobian. This phenomenon was first noticed by Müller in [43] and then generalised by Coifman–Lions–Meyer–Semmes in [14]. The latter paper shows that the Jacobian determinant of maps in ${\dot{W}}^{1,n}(\mathbb {R}^n,\mathbb {R}^n)$ lies in the Hardy space $\mathscr {H}^1(\mathbb {R}^n)$, a fact which was extended in [25] to a large class of polynomials and differential operators. These results are essentially local and, as we are interested in the behaviour of Jacobians on domains, we will need the following global version from [28]:

#### Theorem 2.6

Let $u\in W^{1,n}(\Omega ,\mathbb {R}^n)$ be such that $J u\ge 0$ a.e. in $\Omega$. Then, for any $p>n$,

$$\begin{aligned} \Vert J u \Vert _{L\log L(\Omega )}\le C(\Vert u \Vert _{W^{1-1/p,p}(\partial \Omega )}+\Vert D u \Vert _{L^n(\Omega )}). \end{aligned}$$

There is a more general version of Theorem 2.6 when $J u$ changes sign, but we will not need it here. The space $L\log L(\Omega )$ in the theorem is a particular case of an Orlicz–Zygmund space and it can be defined as the space of those measurable functions $f:\Omega \rightarrow \mathbb {R}$ such that

2.3 $$\begin{aligned} \Vert f \Vert _{L\log L(\Omega )}\equiv \int _\Omega | f(x) | \log \bigg (e+\frac{|f(x)|}{\Vert f \Vert _{L^1}}\bigg ) \,d x<\infty . \end{aligned}$$

The above expression defines a complete norm, although it is not immediate that it satisfies the triangle inequality, see [33, §8]. The standard Luxemburg norm in $L\log L(\Omega )$ is different but equivalent to the one in (2.3); we chose the latter since it is easier to use in calculations. We refer the reader to [47] for the relation between the space $L\log L$ and local Hardy spaces.

## Radial data and radial stretchings

In this section we give a description of the regularity of radial stretchings solving (1.2).

A function $f:B_R(0)\rightarrow \mathbb {R}$ is *radially symmetric* if

$$|x|=|y|\implies f(x)=f(y)$$

and we identify any such function with a function $f:[0,+\infty )\rightarrow \mathbb {R}$ in the obvious way. Under natural assumptions, and when the data is radially symmetric, equation (1.2) admits a unique spherically symmetric solution, that is, a solution of the form $u(x)=\rho (|x|) \frac{x}{|x|}$; we refer to these maps as *radial stretchings*. More precisely, c.f. [6, Lemma 4.1], we have:

### Lemma 2

Let $n>1$ and $1\le p<\infty$. The map $u(x)=\rho (|x|) \frac{x}{|x|}$ is in $W^{1,p}(B_R(0), \mathbb {R}^n)$ if and only if $\rho$ is absolutely continuous on (0, 1) and

$$\begin{aligned} \Vert D u \Vert _{L^p(B_R(0))}^p\approx _n \int _0^R \left( |\dot{\rho }(r)|^p + \left| \frac{\rho (r)}{r}\right| ^p \right) r^{n-1} \,d r<\infty ; \end{aligned}$$

a similar statement holds for $p=\infty$. In this case, for a.e. *x* in $B_R(0)$, and writing $r=|x|$, we have the formulae

3.1 $$\begin{aligned} J u(x)= & {} \frac{1}{{r^{n-1}}}\dot{\rho }(r)\rho ^{n-1}(r), \end{aligned}$$

3.2 $$\begin{aligned} D u(x)= & {} \frac{\rho (r)}{r} Id + \left( \dot{\rho }(r)-\frac{\rho (r)}{r}\right) \frac{x\otimes x}{r^2}. \end{aligned}$$

We also record here the standard notation $\partial _r u(x) \equiv (D u(x))\cdot \frac{x}{r}$; in particular, if *u* is a radial stretching as in Lemma 2, then

3.3 $$\begin{aligned} \partial _r u(x) = \dot{\rho }(r) \frac{x}{r}. \end{aligned}$$

The following proposition follows easily from Lemma 2, see also [52, Theorem 6].

### Proposition 3.1

Let $\Omega =B_R(0)$ and $p\in [1, +\infty ]$. If $f\in L^p(\Omega )$ is radially symmetric and satisfies (1.3) then there is a unique radial stretching *u* with $\rho \ge 0$ solving (1.2). It satisfies

3.4 $$\begin{aligned} \rho ^n(r)=\int _0^r n f(s) s^{n-1} \,d s. \end{aligned}$$

Further, $$u\in W^{1,p}(B_R(0))$$ and

3.5 $$\begin{aligned} \Vert D u \Vert _{L^{p}(B_R(0))} \lesssim _n \frac{\Vert f \Vert _{L^p(B_R(0))}}{(\inf f)^\frac{n-1}{ n}} + R^{\frac{n-1}{p}}\Vert f \Vert _{L^p(B_R(0))}^{\frac{1}{n}}. \end{aligned}$$

Moreover, this inequality is sharp: in general $u\not \in W^{1,q}$ for any $q>p$.

### Proof

The existence and uniqueness of a radial stretching *u* solving (1.2) follows immediately from (3.1).

We turn to the regularity properties and assume that $1\le p<\infty$, as the case $p=\infty$ is similar. Using (3.4) and Jensen’s inequality, we deduce that

$$\begin{aligned} \left| \frac{\rho (r)}{r}\right| ^p\lesssim \left( \fint _{B_r(0)} f(x) \,d x\right) ^\frac{p}{n} \le \frac{1}{r} \left( \int _{B_r(0)} f(x)^p \,d x \right) ^\frac{1}{n}\le \frac{1}{r} \,\Vert f \Vert _{L^p(B_R(0))}^\frac{p}{n} \end{aligned}$$

and therefore, integrating in *r*,

$$\begin{aligned} \int _0^R \left| \frac{\rho (r)}{r}\right| ^p r^{n-1} \,d r \lesssim R^{n-1} \Vert f \Vert _{L^p(B_R(0))}^\frac{p}{n}. \end{aligned}$$

Since *f* satisfies (1.3), we deduce using (3.4) that $(\inf f)^{\frac{1}{n}}\le \rho (r)/r$. Thus, from (3.1), we have

$$\begin{aligned} \dot{\rho }(r) = \frac{r^{n-1}}{\rho ^{n-1}(r)} f(r) \quad \implies \quad \int _0^R |\dot{\rho }(r)|^p r^{n-1} \,d r \lesssim \frac{\Vert f\Vert _{L^p(B_R(0))}^p}{(\inf f)^{\frac{p(n-1)}{ n}}} \end{aligned}$$

and the desired estimate follows from Lemma 2. To see that this inequality is sharp, it suffices to note using (3.1) that for $\delta >0$ there is a constant $C_\delta$ such that $C_\delta f(r) \le \dot{\rho }(r)$ for $r\in (\delta ,R)$. By choosing $f\in L^p(0,R)$ such that $f\not \in L^{q}(\delta ,R)$ for $q>p$ we have $D u\not \in L^{q}(B_R(0))$.

$$\square $$

## Non-solvability of the prescribed Jacobian equation

This section is dedicated to the proof of the main result. We begin by taking $\Omega =B_1(0)$. Throughout this section we fix $c\in (0,1)$.

For $p\in [1,+\infty )$ and $\eta \in [0,1)$, let

4.1 $$\begin{aligned} \begin{aligned} Z_p^{\eta }&\equiv \left\{ f\in L^p(B_1): \fint _{B_1} f \,d x = 1, \, f\ge (1-\eta )c\right\} , \qquad for p>1\\ Z_1^{\eta }&\equiv \left\{ f\in L\log L(B_1): \fint _{B_1} f \,d x = 1, \, f\ge (1-\eta )c\right\} ; \end{aligned} \end{aligned}$$

and note that $X_p\equiv Z^0_p$. We make a few immediate remarks about the sets $Z_p^\eta$:

(i) clearly $$Z_p^{\eta _1}\subset Z_p^{\eta _2}$$ if $$\eta _1<\eta _2$$;
(ii) each set $Z_p^\eta$ is a complete metric space under the distance
  $$\begin{aligned} dist _p(f,g)\equiv {\left\{ \begin{array}{ll} \Vert f -g \Vert _{L^p(B_1)}, &{} p>1\\ \Vert f -g \Vert _{L\log L(B_1)}, &{} p=1 \end{array}\right. }; \end{aligned}$$
(iii) if $c=1$ then the only elements of $X_p$ are functions which are 1 a.e. in $B_1$. Thus we assume throughout this section, without loss of generality, that $c<1$.

We write $B_{Z^\eta _p}(f,\varepsilon )$ for a ball of radius $\varepsilon$ around *f*, under this distance. Given $f\in X_p$ and $\max \{p,n\}\le q$, we consider the *q*-*energy of the datum* *f*, defined by

4.2 $$\begin{aligned} \mathcal {E}_q(f)\equiv \inf \left\{ \int _{B_1}|D v|^{q}\,d x : v solves (1.2)\right\} . \end{aligned}$$

Thus (1.2) admits a $W^{1,q}$ solution if and only if $\mathcal E_q(f)<\infty$. We say that *w* is a *q*-*energy-minimal solution* for *f* if *w* solves (1.2) and moreover

$$\begin{aligned} \mathcal E_q(f) = \int _{B_1(0)} |D w|^q \,d x. \end{aligned}$$

We begin with the following lemma:

### Lemma 3

Fix $1\le p<q<\infty$ and let $\varepsilon ,\eta >0$ be arbitrary. For any $f\in X_p$ there are sequences $f_j\in B_{Z^\eta _p}(f,\varepsilon )$ and $R_j\nearrow 1$ such that

(i) $$dist _p(f_j,f)\rightarrow 0$$ as $$j\rightarrow \infty $$;
(ii) $f_j$ is radially symmetric in $\mathbb A(R_j,1)$;
(iii) if $$u_j$$ is the radial stretching such that $$J u_j=f_j$$ in $$\mathbb A(R_j,1)$$ and $$u_j=id $$ on $$\mathbb S^{n-1}$$, then
  $$\begin{aligned} \lim _{j\rightarrow \infty } \int _{\mathbb A(R_j,1)} |\partial _r u_j(x)|^q\,d x = +\infty . \end{aligned}$$

### Proof

For $\gamma , R \in (\frac{3}{4},1)$, consider the functions

4.3 $$\begin{aligned} f_{\gamma ,R}(x) \equiv {\left\{ \begin{array}{ll} \frac{\gamma ^n}{\fint _{B_R} f \,d x} f(x), &{} 0< |x|< R, \\ \frac{ M [\gamma R + M (|x|-R)]^{n-1}}{|x|^{n-1}}, &{} R \le |x| < 1, \end{array}\right. } \qquad where M \equiv \frac{1-\gamma R}{1-R}. \end{aligned}$$

The choice of *M* ensures that $\fint _{B_1} f_{\gamma , R}\,d x = 1$, since

4.4 $$\begin{aligned} \int _{R}^1 f_{\gamma ,R}(r) r^{n-1} \,d r = \frac{1-(\gamma R)^n}{n}. \end{aligned}$$

Fix $\alpha \in (-\frac{q}{p},-1)$ and choose $\gamma =\gamma (R)$ in such a way that

4.5 $$\begin{aligned} 1-\gamma R = (1-R)^{1+\alpha /q}; \end{aligned}$$

in particular, we have $\gamma (R)\rightarrow 1$ as $R\rightarrow 1$. Take any sequence $R_j \nearrow 1$ and consider the associated numbers $\gamma _j=\gamma _j(R_j)$. We will prove that, for *j* large enough, $f_j\equiv f_{\gamma _j,R_j}$ satisfies the properties above.

Concerning the lower bounds of the sequence, for $r\in (R,1)$, straightforward algebraic manipulations show that

$$\begin{aligned} f_{\gamma ,R}(r) = M \left( \frac{\gamma R + M(r-R)}{r}\right) ^{n-1} \ge 1\times \gamma ^{n-1}; \end{aligned}$$

thus $f_{\gamma ,R}(r)>c$ for $\gamma$ sufficiently close to 1. For $r\in (0,R)$, clearly we have ${f_{\gamma ,R}(r)\ge \gamma ^n c/\fint _{B_R} f\rightarrow c}$ as $\gamma ,R\rightarrow 1$. Hence the lower bounds are satisfied. Moreover, (ii) clearly holds.

For (iii), denote by $u_{\gamma ,R}(x) = \rho _{\gamma ,R}(r) \frac{x}{r}$ the unique radial stretching solving $J u_{\gamma ,R} = f_{\gamma ,R}$ in $\mathbb A(R,1)$ and such that $u_{\gamma ,R}=id$ on $\mathbb S^{n-1}$. By Lemma 2 we find that

4.6 $$\begin{aligned} \rho _{\gamma ,R}(r) =\gamma R + M (r-R), \qquad r\in (R,1] \end{aligned}$$

and so, by (3.3), since $r^{n-1} \,d r\approx \,d r$ for $r\in (\frac{1}{2}, 1)$, and recalling the definition of *M* in (4.3) as well as our choice of $\gamma$ in (4.5),

$$\begin{aligned} \int _{\mathbb {A}(R,1)} |\partial _r u_{\gamma ,R}(x)|^{q} \,d x\approx \int _R^1 |\dot{\rho }(r)|^q \,d r = M^{q} (1-R)= (1-R)^{1+\alpha }. \end{aligned}$$

Thus, since $\alpha <-1$, we see that (iii) also holds.

Hence it remains to prove (i), and we split the proof into two cases.

**Case** $\varvec{p>1}$: Whenever $\gamma ,R \nearrow 1$, we have

$$\begin{aligned} \int _{B_R} |f-f_{\gamma ,R}|^p \,d x = \left| \frac{\gamma ^n}{\fint _{B_R} f \,d x} - 1 \right| ^p \int _{B_R} |f|^p \,d x \rightarrow 0. \end{aligned}$$

Thus, since $\int _{\mathbb {A}(R,1)} |f(x)|^p\,d x\rightarrow 0$ as $R\rightarrow 1$, in order to prove that $f_{\gamma ,R}\rightarrow f$ in $L^p(B_1)$ it suffices to show that, as $\gamma ,R\nearrow 1$,

4.7 $$\begin{aligned} \int _{\mathbb {A}(R,1)} |f_{\gamma ,R}(x)|^p \,d x\rightarrow 0. \end{aligned}$$

For this, note that for $|x|=r\in (R,1)$, since $\gamma R<1$ and $R>\frac{1}{2}$,

$$\begin{aligned} f_{\gamma ,R}(x) \le \frac{M}{r^{n-1}} \le 2^{n-1} \frac{(1-\gamma R)}{1-R}. \end{aligned}$$

Thus, as $r^{n-1} \,d r \approx \,d r$ for $r\in (\frac{1}{2}, 1)$, and using (4.5),

$$\begin{aligned} \int _{\mathbb A(R,1)} |f_{\gamma ,R}(x)|^p \,d x \approx \int _R^1 |f_{\gamma ,R}(r)|^p \,d r \lesssim \frac{(1-\gamma R)^p}{(1-R)^{p-1}}= (1-R)^{\alpha \frac{p}{q} +1}. \end{aligned}$$

Since $\alpha >-\frac{q}{p}$ we have that $\alpha \frac{p}{q} + 1>0$ and so (4.7) is proved.

**Case** $\varvec{p=1}$: As before, we have that, as $\gamma ,R\nearrow 1$,

$$\begin{aligned} \Vert f_{\gamma ,R}-f\Vert _{L\log L(B_R)} = \left| 1-\frac{\gamma ^n}{\fint _{B_R} f\,d x}\right| \Vert f\Vert _{L\log L(B_R)}\rightarrow 0 \end{aligned}$$

and also $\Vert f \Vert _{L\log L(\mathbb {A}(R,1))} \rightarrow 0$ as $R\rightarrow 1$. Thus it suffices to show that ${\Vert f_{\gamma ,R}\Vert _{L\log L(\mathbb {A}(R,1)}\rightarrow 0}$ as $R\rightarrow 1$. We make the simple observation that

$$\begin{aligned} 1-(\gamma R)^n =(1-\gamma R)(1+\gamma R + \dots + (\gamma R)^{n-1})\approx _n (1-\gamma R). \end{aligned}$$

Then, using the estimates on $f_{\gamma ,R}$ from the previous case and (4.4), we obtain

$$\begin{aligned} \Vert f_{\gamma ,R}\Vert _{L\log L(\mathbb A(R,1))}&\approx \int _{\mathbb A(R,1)} f_{\gamma ,R}(x) \log \left( e+\frac{f_{\gamma ,R}(x)}{\Vert f_{\gamma ,R}\Vert _{L^1(\mathbb A(R,1))}}\right) \,d x\\&\lesssim \int _R^1 \frac{1-\gamma R}{1-R} \log \left( e+\frac{1-\gamma R}{(1-R)(1-\gamma ^n R^n)}\right) \,d r\\&\le (1-R)^{\alpha /q+1}\log \left( e+\frac{1}{1-R}\right) . \end{aligned}$$

The right-hand side converges to zero as $R\rightarrow 1$, since $\alpha /q+1>0$. $\square$

Our goal is to show that $\mathcal E_q(f_{\gamma ,R})\rightarrow +\infty$ as $\gamma ,R\rightarrow 1$. The idea is that energy-minimal solutions are controlled by the radial solution.

### Proposition 4.1

Fix $$1\le p<q$$ and suppose that $$v\in W^{1,n}(B_1,\mathbb {R}^n)$$ is a solution of $$J v = f_{\gamma ,R}$$ with $$v=id $$ on $$\partial B_1$$, where $$f_{\gamma ,R}$$ is as in (4.3) and (4.5). There is a constant $$C=C(n,q)>0$$ such that

$$\begin{aligned} \int _{\mathbb A(R,1)} |\partial _r u(x)|^{q}\,d x \le C\int _{\mathbb A(R,1)} |\partial _{r} v(x)|^{q}\,d x, \end{aligned}$$

where $$u=u_{\gamma ,R}$$ is the radial stretching such that $$J u_{\gamma ,R}=f_{\gamma ,R}$$ in $$\mathbb A(R,1)$$ and $$u=id $$ on $$\mathbb S^{n-1}$$.

### Proof

Throughout the proof $\theta$ will denote an element of $\mathbb S^{n-1}$ and we write $r \theta$ for the corresponding element in a sphere of radius *r*.

Since $R > \frac{1}{2}$, from Hölder’s inequality,

$$\begin{aligned} 2^{n-1}\fint _{\mathbb S^{n-1}} \fint _R^1 |\partial _r v(r\theta )|^{q} r^{n-1} \,d r \,d \theta&\ge \fint _{\mathbb S^{n-1}} \fint _R^1 |\partial _r v(r \theta )|^{q} \,d r \,d \theta \\&\ge \left( \fint _{\mathbb S^{n-1}} \fint _R^1 |\partial _r v(r \theta )| \,d r \,d \theta \right) ^{q}. \end{aligned}$$

On the other hand, since $\partial _r u(r \theta ) = M \theta$ for all $r \in (R,1)$, c.f. (4.3) and (4.6), we can estimate

$$\begin{aligned} \left( \fint _{\mathbb S^{n-1}} \fint _R^1 |\partial _r u(r \theta )| \,d r \,d \theta \right) ^{q} =&\fint _{\mathbb S^{n-1}} \fint _R^1 |\partial _r u(r \theta )|^{q} \,d r \,d \theta \\ \ge&\fint _{\mathbb S^{n-1}} \fint _R^1 |\partial _r u(r \theta )|^{q} r^{n-1} \,d r \,d \theta . \end{aligned}$$

Thus, the proposition will be proved once we show that

$$\begin{aligned} \int _{\mathbb S^{n-1}} \int _R^1 |\partial _r v(r \theta )| \,d r \,d \theta \ge \frac{1}{4n^2} \int _{\mathbb S^{n-1}} \int _R^1 |\partial _r u(r \theta )| \,d r \,d \theta \end{aligned}$$

for all $R \in (\frac{1}{2},1)$. Note that $\int _R^1 |\partial _r u(r \theta )| \,d r = (1-R) M = 1 - \gamma R$ for all $\theta \in \mathbb S^{n-1}$.

Let us take the set

$$\Theta \equiv \{\theta \in \mathbb S^{n-1}: r\mapsto v(r\theta ) is absolutely continuous in [R,1]\}.$$

For $\lambda \in (0,1)$ to be chosen later, let

$$\begin{aligned} \Theta _1 \equiv \left\{ \theta \in \Theta : \int _R^1 |\partial _r v(s\theta )| \,d s \le \lambda \right\} ,\quad \Theta _2 \equiv \left\{ \theta \in \Theta : \int _R^1 |\partial _r v(s\theta )| \,d s > \lambda \right\} . \end{aligned}$$

By the ACL property of Sobolev functions, $\mathscr {H}^{n-1}(\Theta )=\mathscr {H}^{n-1}(\mathbb S^{n-1})$ and so the set $\mathbb S^{n-1}\backslash \Theta$ is an $\mathscr {H}^{n-1}$-null set. Fubini’s theorem and the fact that *v* satisfies the Lusin (N) property, c.f. Theorem 2.2, then implies

$$\begin{aligned} \mathscr {L}^n\left( v(\Theta _1\times [R,1])\right) + \mathscr {L}^n\left( v(\Theta _2\times [R,1])\right) = \mathscr {L}^n (v(\mathbb A(R,1)). \end{aligned}$$

By the fundamental theorem of calculus, for $\theta \in \Theta _1$, $r\in [R,1]$,

$$\begin{aligned} 1-|v(r \theta )|\le |v(\theta ) - v(r \theta )| \le \int _r^1 |\partial _r v(s \theta )| \,d s \le \lambda \end{aligned}$$

and thus $v(\Theta _1 \times [R,1])\subset \mathbb A[1-\lambda ,1]$. Combined with the change of variables formula from Corollary 2.3, (4.4) and Bernoulli’s inequality, this estimate yields

$$\begin{aligned} \mathscr {L}^n(v(\Theta _2\times [R,1]))&= \int _{\mathbb A(R,1)} f_{\gamma ,R}(x) \,d x -\mathscr {L}^n(v(\Theta _1\times [R,1])) \\&\ge \omega _n \left[ (1-\gamma ^n R^n)-(1-(1-\lambda )^n)\right] \\&\ge \omega _n \left( 1-n\lambda -\gamma ^n R^n\right) , \end{aligned}$$

since $\mathscr {H}^{n-1}(\mathbb S^{n-1})=n\omega _n$. Moreover, by Markov’s inequality and (4.4),

$$\begin{aligned} \mathscr {L}^n(v(\Theta _2\times [R,1])) =&\int _{\Theta _2\times [R,1]} f_{\gamma ,R}(x) \,d x = \mathscr {H}^{n-1}(\Theta _2)\frac{1-\gamma ^n R^n}{n} \\ \le&\frac{1-\gamma ^n R^n}{n \lambda } \int _{\mathbb A(R,1)} |\partial _r v(r\theta )| \,d r \,d \theta . \end{aligned}$$

Combining the last two estimates and choosing $\lambda =\frac{1-\gamma ^n R^n}{2n}$, we find

$$\begin{aligned} \frac{1}{4} \int _{\mathbb A(R,1)} |\partial _r u(r \theta )| \,d r \,d \theta =&\frac{ n \omega _n (1-\gamma R)}{4} \\ \le&\frac{n \omega _n(1-(\gamma R)^n)}{4}\\ \le&\int _{\mathbb A(R,1)} |\partial _r v(r \theta )| \,d r \,d \theta \end{aligned}$$

since $(\gamma R)^n<\gamma R<1$. The conclusion follows. $\square$

### Remark 1

It is clear from the proof that the boundary condition $$v=id $$ on $$\partial B_1(0)$$ can be weakened to the requirement that $$ v(\theta )\in \mathbb S^{n-1}$$ for $$\mathscr {H}^{n-1}-a.e. $$ $$\theta \in \mathbb S^{n-1}.$$ Note that this condition is independent of the representative of the equivalence class of $$v\in W^{1,q}(B_1(0),\mathbb {R}^n)$$. The argument above carries through simply by replacing the set $$\Theta $$ with $$\Theta \cap \{v(\theta )\in \mathbb S^{n-1}\}$$.

Combining Lemma 3 with Proposition 4.1, we immediately obtain:

### Corollary 4.2

Let $1\le p< q<\infty$ and $n\le q$. For any $\varepsilon ,\eta >0$ and any $f\in X_p$, there is a sequence $f_j\in B_{Z_p^\eta }(f,\varepsilon )$ such that $dist _p(f_j, f)\rightarrow 0$ and $\mathcal E_q(f_j)\rightarrow \infty .$

We are ready to prove our main result, an equivalent formulation of which we restate here for convenience of the reader.

### Theorem 4.3

Fix $0<c<1$ and $1\le p<\infty$ . The set of those $f\in X_p$ such that there is a solution $u\in \bigcup _{n\le q,\, p<q} W^{1,q}(\Omega ,\mathbb {R}^n)$ of (1.2) is meagre in $X_p$.

### Proof

We focus on the case $\Omega = B_1(0)$ first. Fix $\delta >0$ sufficiently small; in particular, $\delta <1-c$ suffices. We replace *c* with $c/(1-\delta )<1$ in the definition (4.1) of $Z_p^\eta$, in order to account for the slightly worse lower bound satisfied by the perturbations of Lemma 3.

Let us first fix *q* such that $n\le q$ and $p<q$. For $k\in \mathbb {N}$ we denote ${Y_k \equiv \{ f \in Z^\delta _p: \mathcal E_q(f)\le k \}.}$ Recall that $E_q(f)$ was defined in (4.2). Note that each $Y_k$ is closed in $Z_p^\delta$. Indeed, given a sequence $f_j\in Y_k$ such that we have ${dist _p(f_j, f)\rightarrow 0}$ for some $f\in Z_p^\delta$, if $v_j$ are energy-minimal solutions corresponding to $f_j$, then from weak compactness there exists a function ${v\in W^{1,q}(B_1,\mathbb {R}^n)}$ such that $v_j\rightharpoonup v$ in $W^{1,q}(B_1,\mathbb {R}^n)$. By the weak continuity of the Jacobian and weak lower semicontinuity of the *q*-Dirichlet energy, it follows that $\mathcal E_q(v)\le \liminf _j \mathcal E_q(v_j)\le k$.

We show that $Y_k$ has empty interior in $Z_p^\delta$. Indeed, take $f_0\in Y_k$ and consider an arbitrary $\varepsilon >0$: we claim that $B_{Z_p^\delta }(f_0,\varepsilon )\not \subset Y_k$. For this, it suffices to show that $B_{X_p}(f_0,\varepsilon )\not \subset Y_k$, since $B_{X_p}(f_0,\varepsilon )\subset B_{Z^\delta _p}(f_0,\varepsilon )$. Any such ball $B_{X_p}(f_0,\varepsilon )$ contains an element $f_1$ which is in $X_p$; thus, by replacing $f_0$ with $f_1$ and shrinking $\varepsilon$ if need be, we can assume without loss of generality that ${f_0\in X_p}$. By Corollary 4.2, there are $f_j \in B_{Z^\delta _p}(f_0,\varepsilon )\subset Y_k$ such that $dist _p(f_j, f)\rightarrow 0$ but $j\le \mathcal E_q(f_j)$. This proves the claim.

Since each set $Y_k$ is closed and has empty interior, the Baire Category Theorem implies that $\bigcup _{k\in \mathbb {N}} Y_k$ is meagre in $Z_p^\delta$. Equivalently, $J (id +W^{1,q}_0(\Omega ,\mathbb {R}^n))$ is meagre in $Z_p^\delta .$ For $p\ge n$, we have that

$$\begin{aligned} J \biggr (id +\bigcup _{n\le q,\, p<q} W^{1,q}_0(\Omega ,\mathbb {R}^n)\biggr ) = \bigcup _{j=1}^\infty J \left( id +W^{1,q_j}_0(\Omega ,\mathbb {R}^n)\right) , \qquad q_j\searrow p, \end{aligned}$$

and the right-hand side is a countable union of meagre sets, hence meagre, so the conclusion follows. The case $p<n$ is identical.

We conclude the proof by considering the case where $\Omega$ is a general smooth and uniformly convex domain. In this case there is a smooth diffeomorphism ${\varphi :\overline{\Omega }\rightarrow \overline{B_1(0)}}$ with $J \varphi =\omega _n/|\Omega |$, see for instance [22, Theorem 5.4] for a more general version of this fact; the existence of such a map can also be deduced from Caffarelli’s regularity theory for the Monge–Ampère equation, see e.g. [18, Theorem 3.3]. We can now identify every map $u:\Omega \rightarrow \mathbb {R}^n$ with a map $v:B_1(0)\rightarrow \mathbb {R}^n$ with the same regularity, through $v\equiv \varphi \circ u \circ \varphi ^{-1}$ and moreover, when $x\in \partial B_1(0)$, we have $v(x) = x$, as $\varphi (\partial \Omega )=\partial B_1(0)$. Since $J v = J u \circ \varphi ^{-1}$, the general statement follows from the case $\Omega =B_1(0)$. $\square$
